# Authority and Teaching

**Published:** 1908-03-21

**Authors:** 


### The Hospital

A Journal of

The Medical Sciences and Hospital Administration.

New Series. No. 53, Vol. II. [No. 1125, Vol. XLIII.] SATURDAY, MARCH 21, 1908.

### AUTHORITY AND TEACHING.

"Authority," said Boerhaave, "is nothing to the physician, unless it is consonant with experience." The plea is often made that dogmatism is a necessity in clinical teaching, but, like most of such statements, it is a fallacious argument, for it presupposes, to a certain extent at least, an inability on the part of the student to reason for himself. On the one hand we have the teachers of the profession declaring that vivisection, for example, is a necessity in routine teaching of students in preliminary subjects in order to enable them to judge for themselves the truth or the exposure of this claim or that theory: on the other we have the claim, put forward in the preface of nearly every text-book, that a moderate insistence on authority is absolutely essential. The two positions are somewhat inconsistent with each other, though less so, perhaps, to the professional than to the lay mind. Every practitioner knows that there are certain statements which are acceptable, not because they can be proved by each of us to be true, but because they have been demonstrated in a fashion that leaves no room for doubt on the part of reasonable men. That extirpation of the pancreas leads to glycosuria, for instance, as was demonstrated by Von Mering and Minskowski, is a statement which few of us will cavil at, though as few, perhaps, have seen the experiment actually performed. The student accepts the statement as a fact while he is yet in the physiological laboratory, just as readily as he accepts Brown Sequard's paralysis though he has never seen a hemi-section of the cord performed, or the circulation of the blood though he has never troubled to repeat Harvey's experiments. Later on, when he reaches the wards, he has unlimited scope for demonstrating to himself the truth of these hypotheses by observation and clinical study. The argument may, it is true, be carried too far. It is permissible to believe that the actual ocular demonstration of the functions of muscle by electrical stimulation, of nerve by stimulation, impresses upon the student certain elementary facts far better and in a manner far clearer than the mere wordy recital of such functions would have done. Therein, and therein alone, lies, as we take it, the only justification for the performance of certain experiments in the class-room. But it may reasonably be argued that if this principle holds good with elementary facts it should also hold good with major facts. To be consistent, the physiological lecturer should perform each and every experiment that has been done to demonstrate the truth or fallacy of certain theories in order to impress upon his pupils the value of what has been gained by research. That is a position which is untenable in practice, however fair it may be in theory, and a consideration of the whole question forces upon us the truth of the statement that in all medicine authority must of necessity maintain a high place. It is an interesting fact that those who most lay stress upon the necessity for dogmatism in teaching at the same time proclaim their abhorrence of empiricism. Dogmatism is essentially a system of rationalism, the sect of which Boerhaave was a shining light and which nowadays numbers many adherents. The dogmatist, in the words of Dr. Gee, seeks to understand what medicine is, what disease. He formulates systems, builds up theories, experiments, and clinches by authority what he fails to find out by deduction. It is the sceptical dogmatist that tells us that formates have no therapeutical utility; that camphor is useless as a stimulant because it does not raise the blood-pressure; that strychnine is an abomination in cases of shock; and that (though the sect, as a whole, would resent this example) like cures like. Each one of us, in his student days at least, was possibly an intolerant dogmatist, prone to inquisitorial ways, to regard as heresy any dissonance from the views of old-established authority. Had Thackeray interested himself in medical sects, assuredly he would have found a place for the dogmatist in his calendar of snobbery.

Only when he observes for himself does the medical student—and the true physician is a student to the end of his days—find out the inconsistencies and the absurdities of the systems, and come to range himself among the empirics. Few of us acknowledge that we have a sneaking distrust of authority, and an equally shamefaced predilection, fostered by ward and practical clinical experience, in favour of empiricism. Where authority is in accord with experience, we are willing to trust it. The new drug is adopted because somebody used it in a particular case with beneficial results, not merely because someone else read a paper before this or that society and pointed out its action on the unstriated muscle of Rana esculenta or on the venom of Naje haje. This in itself is an admission that authority, for all the distrust that it evokes, has still a great deal of weight with us in treatment, diagnosis, and prophylaxis. The authoritative enunciation of certain facts will always have a place in our teaching, for without it teaching would lose half its value and become a mere mechanical task of affording examples. The true way of combining the two, example and precept, is the way which is followed by most of our best clinical teachers—a limited insistence upon authority, but, more than that, the constant and unwearied demonstration to the student of methods—methods of observation, of thought, of selection, compilation, and last, but by no means least, of discrimination and application of what has been learned.

